# The persuasive essays for rating, selecting, and understanding argumentative and discourse elements (PERSUADE) corpus 1.0

**DOI:** 10.1016/j.asw.2022.100667

**Published:** 2022-10

**Authors:** Scott A. Crossley, Perpetual Baffour, Yu Tian, Aigner Picou, Meg Benner, Ulrich Boser

**Affiliations:** Department of Special Education Peabody College Vanderbilt University 110 Magnolia Circle Nashville, TN 37212

**Keywords:** Persuasive writing, Corpus, Discourse elements

## Abstract

This paper introduces the Persuasive Essays for Rating, Selecting, and Understanding Argumentative and Discourse Elements (PERSUADE) corpus.The PERSUADE corpus is large-scale corpus of writing with annotated discourse elements. The goal of the corpus is to spur the development of new, open-source scoring algorithms that identify discourse elements in argumentative writing to open new avenues for the development of automatic writing evaluation systems that focus more specifically on the semantic and organizational elements of student writing

## Highlights

• We introduce the PERSUADE corpus.
• The corpus contains over 25,000 essays with annotated discourse elements.
• The PERSUADE corpus can be used to assess writing quality.

## 1 Introduction

Writing is an essential skill for college and career success. Still, many students struggle to produce writing that meets college and career standards (NCES, 2012). One way to help students improve their writing is to provide students with more opportunities to write and receive feedback on their writing (Graham & Perin, 2007). However, assigning more writing to students places a burden on teachers to generate timely feedback. One potential solution is the use of automated writing evaluation (AWE) systems, which can evaluate student writing and provide feedback independently. These kinds of systems can encourage students to write and revise more frequently and reduce the amount of time that teachers spend grading (Strobl et al., 2019).

The feedback algorithms found in AWE systems rely on corpora of essays that have been generally hand-coded by raters for specific elements related to writing. These elements may include holistic scores of writing quality (Shermis, 2014), analytic scores of quality that focus on specific text elements like organization, grammar, or vocabulary use (Crossley & McNamara, 2010), or annotations of argumentative elements like claims (Stab & Gurevych, 2017). However, large corpora that are annotated for argumentative elements are non-existent, thus making it difficult for AWE systems to provide accurate and reliable feedback on important elements of writing success.

Here, we introduce the Persuasive Essays for Rating, Selecting, and Understanding Argumentative and Discourse Elements (PERSUADE) corpus. The PERSUADE corpus is an open-source corpus comprising over 25,000 essays annotated for argumentative and discourse elements and relationships between these elements. In addition, the PERSUADE corpus includes detailed demographic information for the writers.

Our goal in releasing the corpus is to spur the development of new, open-source scoring algorithms that identify discourse elements in argumentative writing. Because the PERSUADE corpus also includes detailed demographic information, developed algorithms can also be assessed for potential bias to ensure they do not favor one population over another. Once developed, algorithms can be included in AWE systems to provide more pinpointed feedback to writers about their use of argumentative and discourse elements. Such feedback would open new avenues for the development of AWE systems that focus more specifically on the semantic and organizational elements of student writing.

The PERSUADE corpus was pulled from a larger corpus of student writing (N = ~500,000). The PERSUADE corpus comprises two sub-corpora consisting of source-based essays (n = 12,875) and independent essays (n = 13,121). Source-based writing requires the student to refer to a text while independent writing excludes this requirement. The source-based set was derived from seven unique writing prompts and related sources. The writing reflects students in grades 6 through 10. The independent set reflects writing where background knowledge of the topic was not a requirement, and no sources were required to produce the texts. The independent sub-corpus was collected from students in grades 8 through 12, and the collection was derived from eight unique writing prompts. All prompts and sources are available within the PERSUADE corpus.

The PERSUADE corpus was limited to essays with a minimum of 150 words of which 75% had to be correctly spelled American English words. These filters were used to ensure appropriate coverage of argumentative and discourse elements in the texts as well as to ensure the essays contained enough language from which to develop natural language processing (NLP) features to inform algorithm development (Crossley, 2018). Additionally, the filters help to confirm that the essays were written in English and that the essays did not contain a large amount of gibberish. Descriptive statistics for number of words, number of sentences, and number of paragraphs per essay are reported in Table 1.

**Table 1:** Descriptive statistic for PERSUADE corpus.

	Mean	SD	Median	Range
Number of words	402.31	188.38	364.00	2003.00
Number of sentences	20.56	9.76	19.00	132.00
Number of paragraphs	5.52	3.81	5.00	68

**Fig. 1:**
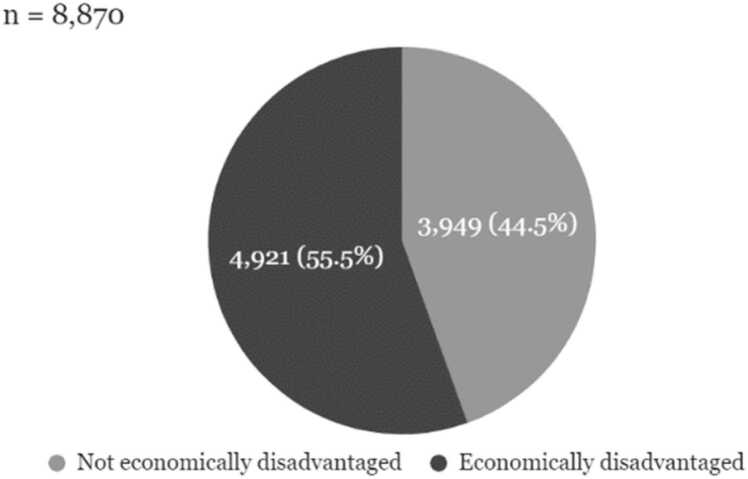
Economic composition of PERSUADE corpus authors, text-dependent essays.

**Fig. 2:**
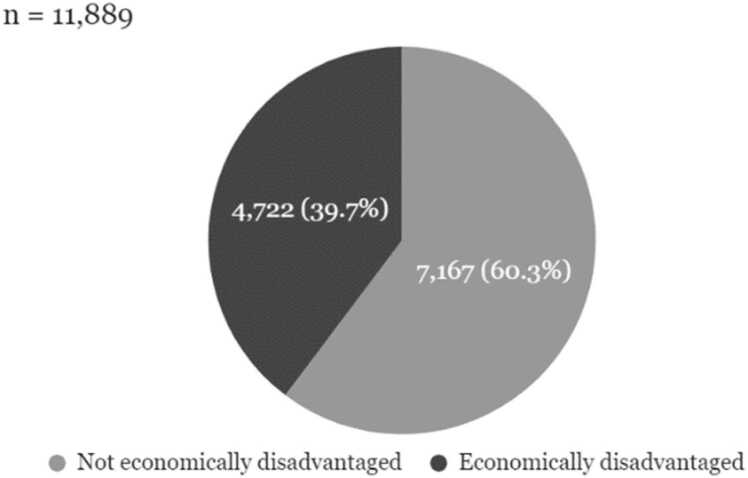
Economic composition of PERSUADE corpus authors, independent essays.

**Fig. 3:**
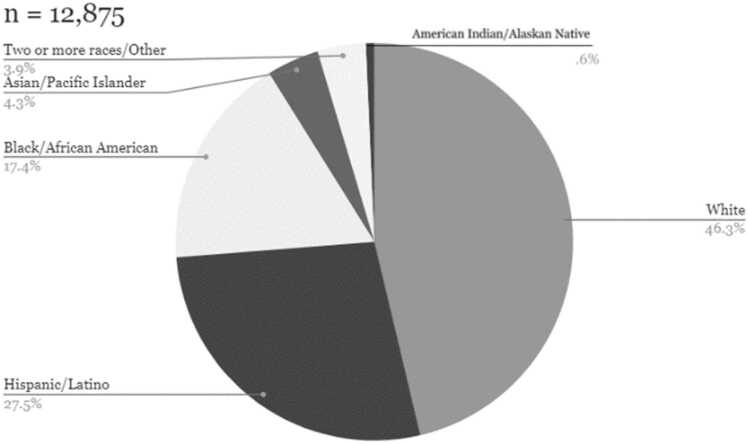
Racial/ethnic composition of PERSUADE corpus authors, text-dependent essays.

**Fig. 4:**
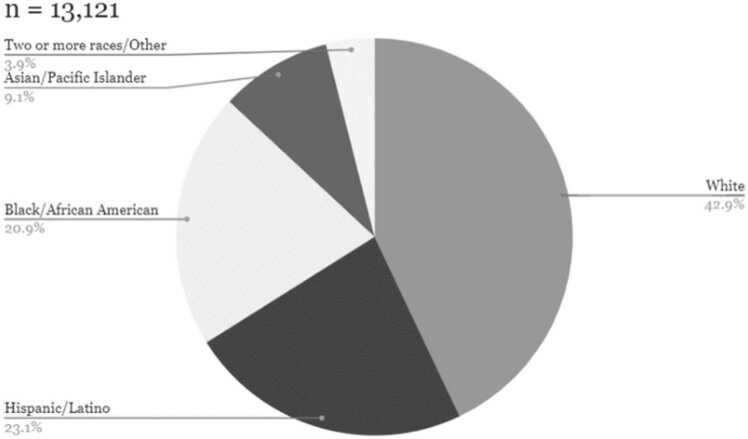
Racial/ethnic composition of PERSUADE authors, independent essays.

The PERSUADE corpus was selected to reflect a range of writing from diverse student populations that was representative of the writing population in the United States. All essays in the PERSUADE corpus are linked to information on the student’s gender and race/ethnicity. A subset of the corpus (n = 20,759) also contains data on student eligibility for federal assistance programs such as free or reduced-price school lunch, Temporary Assistance for Needy Families, and the Supplemental Nutrition Assistance Programs, which we broadly define as economic disadvantage. A large sub-sample of the essays in the corpus also includes information on English Language Learner status (n = 24,787) and disability status (n = 24,828). The racial, gender, and economic composition of the corpus collection closely resembles the U.S. secondary public school population using data from the National Center for Education Statistics as a benchmark. Descriptive statistics on demographic representation in terms of economic disadvantage and race/ethnicity are reported in Fig. 1, Fig. 2, Fig. 3, Fig. 4.

Each essay in the PERSUADE corpus was human annotated for argumentative and discourse elements as well as relationships between argumentative elements. The corpus was annotated using a double-blind rating process with 100% adjudication such that each essay was independently reviewed by two expert raters and adjudicated by a third expert rater. All ratings were completed by an educational consulting firm in the United States and by raters with at least two years of experience. Prior to norming, raters received anti-bias training and anti-bias strategy instruction that was designed to address issues of bias that occur during scoring and are inherent to the use of standardized rubrics (Warner, 2018). Raters used an annotation platform provided by a third-party commercial partner that allowed raters to highlight text segments of an essay, assign a discourse element category to each segment, and provide effectiveness ratings and hierarchical relations for that segment. Raters were trained on each prompt separately and on independent and source-based essays separately. Raters were provided with bridge sets for each prompt that included essays of varying quality. Ratings were spot-checked throughout the process to ensure rater accuracy.

The annotation rubric was developed to identify and evaluate discourse elements commonly found in argumentative writing. The rubric was developed in-house and went through multiple revisions based on feedback from two teacher panels as well as feedback from a research advisory board comprising experts in the fields of writing, discourse processing, linguistics, and machine learning. The discourse elements chosen for this rubric come from Nussbaum, Kardash, and Graham (2005) and Stapleton and Wu (2015). Both annotation schemes are adapted or simplified versions of the Toulmin argumentative framework (1958). Elements scored and brief descriptions for the elements are provided below.

*Lead.* An introduction that begins with a statistic, a quotation, a description, or some other device to grab the reader’s attention and point toward the thesis.

*Position.* An opinion or conclusion on the main question.

*Claim*. A claim that supports the position.

*Counterclaim.* A claim that refutes another claim or gives an opposing reason to the position.

*Rebuttal.* A claim that refutes a counterclaim.

*Evidence.* Ideas or examples that support claims, counterclaims, rebuttals, or the position.

*Concluding Statement.* A concluding statement that restates the position and claims.

**Fig. 5:**
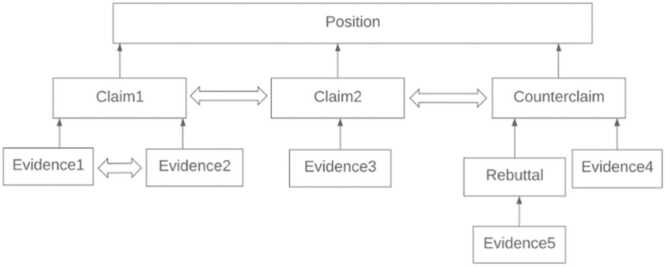
Prototypical Diagram for Relations Among Argumentation Elements.

Relationships between the argumentative elements are illustrated through a hierarchical organization inspired by Rhetorical Structure Theory (Mann & Thompson, 1988) and general tree structures. The purpose of the relationships is to examine organization and coherence among argumentative elements at the text level. Each argumentative element is marked as a parent, child, or sibling of another element. For example, a claim that supports a position is annotated as the child of the position. Supporting evidence for the claim is annotated as a child of the claim. If two pieces of evidence are provided for a claim, they will both be labeled children of the claim and siblings of one another. An overview of these relationships is depicted in Fig. 5.

## 2 Learning objectives and related research

Argumentation can be viewed as a logical appeal that involves stating claims and offering support to justify or refute beliefs to influence others (Newell, Beach, Smith, & VanDerHeide, 2011). The ability to persuade with good argumentation skills lies at the core of critical thinking and has long been valued in personal, professional, and academic contexts. Given the important role of argumentation in students’ cognitive development and academic learning, the K-12 Common Core State Standards (2010) highlights the cultivation of argumentation skills in writing instruction and stipulates that students need to achieve proficiency in using valid reasoning and relevant and sufficient evidence to support claims. However, many students in the U.S. struggle to construct a solid argument in writing due to its cognitively demanding nature. According to the 2012 NAEP Writing Report Card, only about 25% of students' argumentative essays were competent. Thus, systematic analysis of the arguments in students' essays informed through in-depth understanding of informal reasoning and written argumentation affords tremendous pedagogical values.

Identifying the generic elements that compose an argument is the starting point for analyzing arguments in students' essays. Although argument has been interpreted and conceptualized differently depending on specific sets of theoretical assumptions (Newell et al., 2011), research has generally indicated that Toulmin (1958) model of informal argument and its variations are effective in capturing the type of organizational structures in students' argumentative writing (e.g., Knudson, 1992). Toulmin's model of argument revolves around three key elements: a *claim*, or the assertion to be argued for, *data* that provide the supportive evidence (empirical or experiential) for the claim, and a *warrant* that explains how the data support the claim. To capture the different aspects related to the nature of human reasoning, Toulmin also added three other argument elements: *backing* that affords justifications for the warrant, *qualifiers* to signal the strength of the argument, and *rebuttals* that denote exceptions to the elements of the argument.

Whereas Toulmin's model lends itself well to the analysis and construction of single claims, it is less helpful to deal with the structure of arguments at the macro level (Wingate, 2012). Therefore, modified versions of Toulmin’s model have been developed to attend to the macrostructure of written argumentation. For instance, Nussbaum et al. (2005) adapted Toulmin's model to feature an opinion or a conclusion on the main question (*final claim*) which is usually supported by one or more reasons (*primary claims*) or claims (*rebuttals*) refuting some potentially opposing opinions (*counterclaims*). Nussbaum et al. also included into the model *supporting reasons or examples* used to back up the stated claims. Similarly, Qin and Karabacak (2010) used a coding scheme based on Toulmin's model that comprised six elements: *claim*, *data*, *counterargument claim*, *counterargument data*, *rebuttal claim*, and *rebuttal data* to identify argument elements in argumentative essays.

There are many problems with relying solely on prototypical argumentative discourse schema like those laid out by Toulmin. One problem is that such approaches are not based on theories of text analysis and may thus lack construct relevancy (Azar, 1999). Additionally, discourse schemas do not show relations at the text level, giving the impression that essays are static and making it difficult to understand how argumentative elements can shape an entire text (Freeman, 2011). To address these problems, Mann and Thompson (1988) developed Rhetorical Structure Theory (RST) which helps to arrange and connect parts of a text type to construct a whole. RST does this by focusing on relationships between discourse elements to demonstrate how a whole text functions. Specifically, discourse elements are connected through a small set of rhetorical relations that break texts into segments and develops relationships between segments to connect them coherently (Azar, 1999). Azar concluded that RST was a useful tool for modeling argumentative text that complemented Toulmin’s approach. Green (2010) also adopted RST to model argumentative texts by using hierarchical trees to identify how evidence can link a claim with its argument and how a background relationship can link evidence with its warrant.

## 3 Connections

There are few currently available corpora that focus on assessing argumentation in persuasive writing and none that include rhetorical features like leads or concluding summaries. While existing corpora provide annotations for argumentative elements, the corpora are small, do not contain detailed argumentative features, do not focus on argumentative relationships, and do not contain demographic information.

The two best known corpora annotated for argumentative elements were released by Stab and Gurevych, 2014, Stab and Gurevych, 2017. Their initial corpus released in 2014 was small and consisted of 90 essays. The argument components annotated include *major claims*, *claims*, and *premises*. Major claims referred to sentences that directly expressed the general stance of the author that was supported by additional arguments. Claims were the central component of an argument, and premises were reasons that supported the claims. Stab and Gurevych also annotated the relationships between premises and major claims or claims in terms of whether they supported the claims or not. A follow up corpus followed the same annotation procedure and was released in 2017. This corpus contained 402 argumentative essays written by students (including the original 90 essays in the 2014 corpus). Both corpora were publicly released in order to increase access to annotated instances of argumentation in essays.

## 4 Limitations and future steps

The PERSUADE corpus was the foundation for the Feedback Prize competition hosted by Kaggle, an online community of data scientists. The Feedback Prize sought to develop machine learning models to best classify discourse elements in the PERSUADE corpus. Over 2000 teams participated, and the twelve top teams shared $160,000 in prize money. The winning model reported a classification accuracy for argumentative and discourse elements of just over 75%. All winning algorithms and the training portion of the PERSUADE corpus are freely available on the Feedback Prize Kaggle website (https://www.kaggle.com/c/feedback-prize-2021).

As a large-scale, open-sourced corpus of annotated discourse elements, the PERSUADE is unparalleled. However, it does have limitations. For instance, the corpus only focuses on 6–12th grade writers, leaving out younger writers developing proficiency and older, more proficient writers. The corpus also has a limited number of prompts (N = 15) and the current release of the PERSUADE corpus does not include quality ratings for the discourse elements or the essay as a whole. Lastly, the corpus only focuses on independent and integrated writing tasks (i.e., argumentative essays). Argumentative essays are overrepresented in secondary schools and first-year composition courses, especially when compared to the types of writing (e.g., explanatory writing) that students are exposed to in their post-secondary courses (Aull and Ross, 2020, Aull, 2019). In this sense, the PERSUADE corpus may lead to increased generalizations about the narrowness of academic writing that favors argumentation over other types of persuasion. This may be compounded if models to classify argument types based on PERSUADE are incorporated into AWE systems as planned. AWE systems, which have wide uptake, have the potential to further popularize the notion that academic writing is best represented through argumentation. Thus, future corpora would benefit from the inclusion of writing samples from compare-and-contrast essays, research reports, and analysis papers.

While the PERSUADE corpus was designed for machine learning, it is available to anyone, and we envision it will be used by writing researchers interested in both qualitative and quantitative methodologies. We also presume that the PERSUADE corpus could be used for other pedagogical applications including student-centered assessment, the development of heuristics for explicit genre knowledge, descriptive feedback in peer-reviews, and other uses developed by classroom teachers and writing program administrators.

Our next steps are to collect quality ratings for the individual discourse elements and the essays. We will then host additional Kaggle competitions to develop algorithms to predict discourse element quality and holistic essay score. Once those competition are completed, the entire PERSUADE corpus will be released publicly on Kaggle and other websites.
